# Changing Self‐Assembly Through Degradation: Phenyl Vinyl Ketone Polymer Nanoparticles Under Light

**DOI:** 10.1002/anie.202514545

**Published:** 2025-09-08

**Authors:** M. A. Sachini N. Weerasinghe, Parker Anthony McBeth, Michelle C. Mancini, Dominik Konkolewicz

**Affiliations:** ^1^ Department of Chemistry and Biochemistry Miami University 651 E High St Oxford OH 45056 USA

**Keywords:** Photochemistry, PISA, Self‐assembly, Photodegradation

## Abstract

Photodegradable nanoparticles with sphere, worm, and vesicle morphologies were synthesized following polymerization induced self‐assembly (PISA), incorporating a photoresponsive phenyl vinyl ketone (PVK) block and a nonphoto responsive 2‐hydroxypropyl methacrylamide (HPMA) block. The photodegradation of nanoparticles under UV revealed that the initial shapes of sphere and vesicle particles are retained even until 7 h and after 24 h of photo‐induced degradation, respectively, despite a significant reduction in molecular weight (*M_n_*). This could be due to the assembly of degraded PVK fragments in the hydrophobic region, maintaining the relative hydrophilic to hydrophobic ratio. However, worm nanoparticles exhibited a fast morphology reversal after 2 min of degradation, yielding sphere nanoparticles. Therefore, photo responsive PVK nanoparticles with morphology and conversion‐dependent degradation behavior were explored. Furthermore, undegraded and degraded nanoparticle coatings exhibited different surface properties, determined by contact angle measurements, with both morphology and degradation impacting the surface properties. Finally, the PVK nanoparticles could encapsulate molecules and release them upon degradation. Therefore, this study can ultimately be applied to numerous fields due to the potential uses of degradable polymers in various material systems, especially for the controlled release of agrochemicals, cleaning agents, or antifungal agents, as well as to enable surface modifications upon degradation.

## Graphical abstract

Photodegradable nanoparticles with sphere, worm, and vesicle morphologies are created via PISA using photo responsive PVK and non‐responsive HPMA blocks. Under UV light, worms quickly transform into spheres, while spheres and vesicles retain their shapes despite chain scission. Degradation also tailors surface properties and enables controlled release, offering promising applications in smart coatings, responsive materials, and targeted delivery systems.

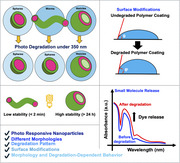

## Introduction

Due to their distinctive and remarkable properties, the preparation of nanoparticles with different morphologies is essential for various applications.^[^ ^1^, ^2^, ^3^, ^4^ ^]^ In polymer‐based nanoparticles, several common morphologies exist, including spheres, worms, and vesicles.^[^ ^5^, ^6^, ^7^ ^]^ As the length of the core‐forming block increases, the morphology transitions from spheres to worms, and then from worms to vesicles.^[^ ^8^, ^9^ ^]^ Sphere‐shaped nanoparticles have higher cellular uptake compared to worm‐shaped nanoparticles.^[^ ^10^, ^11^, ^12^, ^13^ ^]^ Worm nanoparticles have higher viscosity and aspect ratio.^[^ ^14^, ^15^ ^]^ Therefore, it can be used to achieve longer blood circulation in drug delivery applications^[^ ^16^, ^17^, ^18^ ^]^ and to use as catalysts^[^ ^4^ ^]^ or viscosity modifiers.^[^ ^19^, ^20^, ^21^, ^22^ ^]^ Vesicles have efficient encapsulating ability for both hydrophilic and hydrophobic agents in their hydrophobic and hydrophilic compartments.^[^ ^2^, ^3^, ^12^, ^23^, ^24^, ^25^ ^]^ Recently, a plethora of examples of synthesizing polymer nanoparticles with different morphologies have been discussed. Several techniques, such as solvent evaporation,^[^ ^26^, ^27^ ^]^ nanoprecipitation,^[^ ^27^, ^28^ ^]^ or polymerization induced self‐assembly method (PISA),^[^ ^29^, ^30^ ^]^ have been applied.

**Figure 1:**
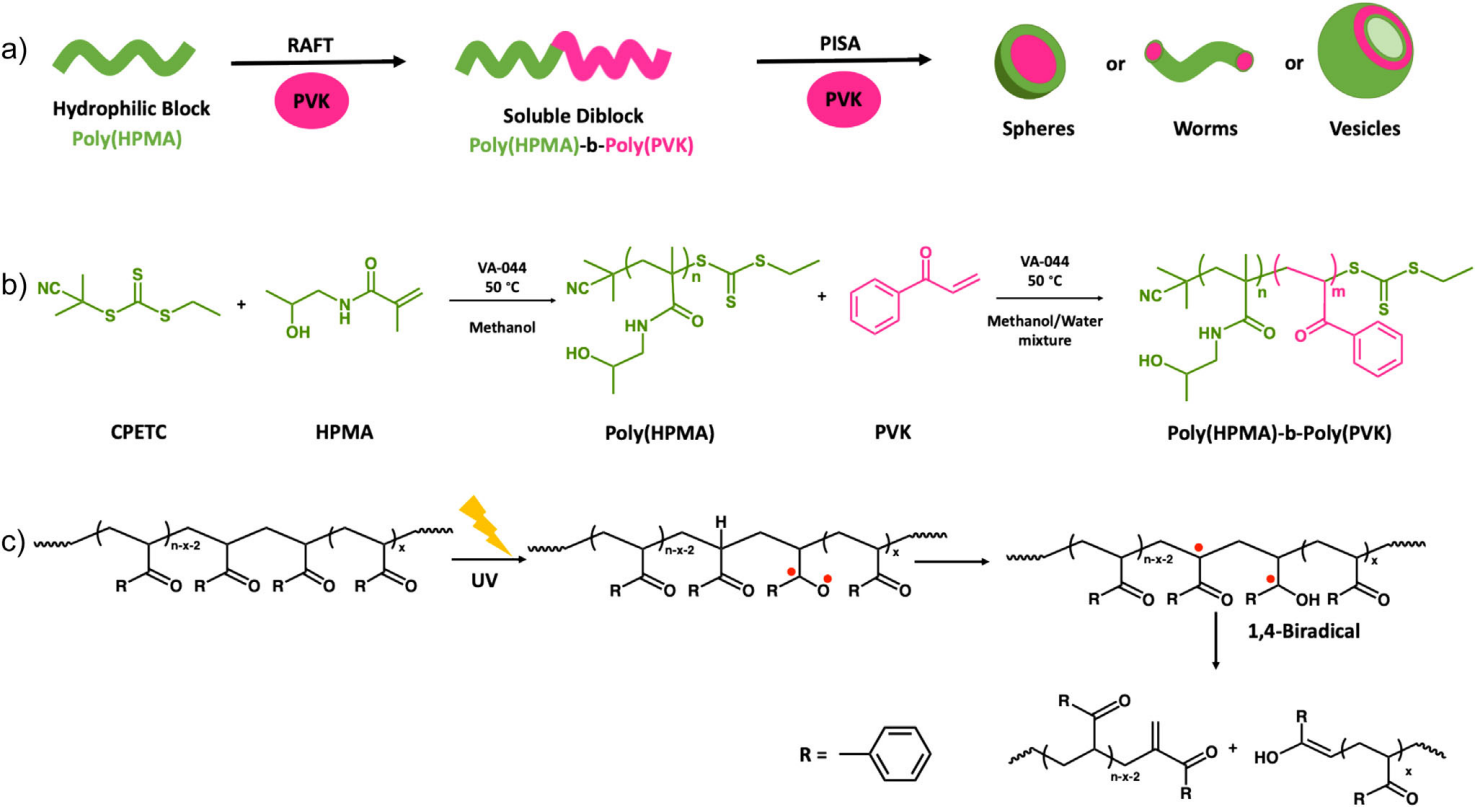
a) Schematic representation of PISA. b) Polymerization reaction of the first hydrophilic poly(HPMA) followed by chain extension reaction in the presence of PVK monomer to obtain poly(HPMA)‐b‐poly(PVK). Conditions: RAFT polymerization of poly(HPMA)‐ In the presence of a molar ratio of HPMA:CPETC:VA‐044 = 25:1:0.2 and methanol as the solvent at 50 °C. Chain extension‐ In the presence of a molar ratio of PVK:poly(HPMA):VA‐044 = 400:1:0.2, methanol/water mixture as the solvent (e.g., 50% MeOH, 60% MeOH, and 75% MeOH), and different solid concentrations (10, 20, and 30 w/w%) at 50 °C. c) Schematic representation of the Norrish type II reaction under UV irradiation, generating radicals on the PVK polymer backbone and enabling main‐chain cleavage.

Among those techniques, PISA is recognized as a versatile and robust one‐pot technique to synthesize nanoparticles with different morphologies and sizes.^[^ ^29^, ^30^ ^]^ In PISA, a soluble polymer block is chain extended in the presence of a second monomer and a suitable solvent, where the growing block gradually makes an insoluble polymer which then self‐assembles into nanoparticles as illustrated in Figure 1a.^[^ ^8^, ^29^, ^30^ ^]^ During the chain extension, spheres appear as the primary morphology. Subsequently, the fusion of spheres leads to the formation of linear cylinders or worms. Finally, vesicles develop after passing the stages of linear worms, branched worms, octopi, and jellyfish.^[^ ^9^ ^]^ Several parameters, including the chain length of the hydrophilic or hydrophobic block, solvent composition, and solid concentration, can be varied to obtain nanoparticles with a desired size and shape as these characteristics are controlled by the relative volume ratio of the hydrophilic and hydrophobic blocks.^[^ ^29^, ^30^ ^]^

A range of techniques is available for the fabrication of polymer nanoparticles and for inducing morphological transitions in pre‐existing nanostructures. This can be performed by changing the system via the addition of salts, ^[^ ^31^, ^32^ ^]^ acids,^[^ ^33^ ^]^ bases,^[^ ^33^ ^]^ small organic molecules,^[^ ^34^ ^]^ or using stimuli responsive blocks^[^ ^33^, ^35^, ^36^ ^]^ in the nanoparticle preparation. The main benefit of this approach is the convenience of obtaining different shape nanoparticles using a single system rather than synthesizing diblock copolymers with different chain lengths or optimizing several experimental parameters to obtain different shapes. Materials that are sensitive to stimuli such as light, temperature, or pH have gained recent attention because of their ability to introduce various interesting properties upon the application of the stimuli.^[^ ^37^, ^38^, ^39^ ^]^ Incorporation of a responsive block in self‐assembled nanoparticles can enable these objects to undergo morphological transitions.^[^ ^33^, ^35^, ^36^ ^]^ Despite the potential of existing systems to change their self‐assembly upon stimulus, existing reports often involve temperature variations or the addition of foreign substances into the system.

Photo responsive materials have gained immense attention due to their ability to perform a reaction under mild conditions without requiring reagents or generating chemical byproducts from the stimulus.^[^ ^40^, ^41^, ^42^ ^]^ Therefore, light‐driven systems are preferable in various medical applications, including drug delivery systems, tissue engineering, polymeric photosensitizers in photodynamic therapy, surgical adhesives, etc.^[^ ^43^, ^44^, ^45^ ^]^ Photo responsive nanoparticles with photocleavable,^[^ ^46^, ^47^ ^]^ photoisomerizable,^[^ ^48^, ^49^, ^50^ ^]^ or photo decrosslinking mechanisms,^[^ ^51^, ^52^ ^]^ have been studied. The reported systems typically bear coumarin,^[^ ^51^ ^]^ o‐nitrobenzyl,^[^ ^53^, ^54^ ^]^ azobenzene,^[^ ^48^, ^55^, ^56^ ^]^ or spiropyran^[^ ^49^, ^50^, ^57^ ^]^ groups as the photo responsive group. Typically, specific synthetic steps or techniques are required to attach functional groups to monomers before preparing polymer nanoparticles. In contrast, vinyl ketone polymers can readily impart dramatic photo responsive properties to nanoparticle systems due to their intrinsic UV‐induced backbone degradability, enabling changes in polymer structure and material properties.^[^ ^58^, ^59^ ^]^ Vinyl ketone polymers undergo photodegradation under UV light through a proposed Norrish type II mechanism.^[^ ^58^, ^60^, ^61^, ^62^ ^]^ Previously, studies of vinyl ketone monomers and polymers identified these materials light‐dependent polymerization and degradation kinetics.^[^ ^58^, ^60^, ^63^, ^64^ ^]^ This study explores the synthesis of phenyl vinyl ketone (PVK) nanoparticles via PISA, their potential morphological transformations through the photodegradation of PVK block under UV, surface modifications made by undegraded and degraded nanoparticle coatings, and their ability to release small molecules upon degradation. Such photodegradability would give an added value to a nanoparticle system, enabling a wide range of potential applications, especially considering the high demand for degradable polymers.^[^ ^65^, ^66^, ^67^ ^]^

## Results and Discussion

In this PISA system, the hydrophilic polymers based on poly(2‐hydroxypropyl methacrylamide) (poly(HPMA))^[^ ^68^ ^]^ and hydrophobic polymers based on poly(PVK) were selected. First, the hydrophilic macro‐chain transfer agent (CTA) based on poly(HPMA) was synthesized by reversible addition fragmentation chain transfer polymerization (RAFT) of HPMA in methanol at 50 °C, following the modified procedure.^[^ ^69^ ^]^ RAFT polymerization yielded a well‐controlled polymer with a narrow molecular weight distribution (*M_n _* = 5100, *M_w_/M_n _* = 1.15) with >95% monomer conversion.

Throughout the study, the total target chain length was kept constant by using a hydrophilic block with a degree of polymerization of 25 (DP = 25) and a hydrophobic block with a DP of 400. However, chain extension polymerization was carried out by varying the ratio of methanol to water in the solvent system (methanol:water = 50:50 (50% MeOH), methanol:water = 60:40 (60% MeOH), and methanol:water = 75:25 (75% MeOH)) and total solid concentration (10, 20, and 30 w/w%). For simplicity, only the characterizations of the 50% MeOH system with all three solid concentrations were included in the main text, though the characterizations of 60% MeOH and 75% MeOH systems were included in the Supporting Information. These three systems explored the degradation behavior of PVK nanoparticles with sphere, worm, and vesicle shapes to understand morphological transitions or stability of those shapes under light in this model system. As illustrated in Figure 1b, the chain extension of poly(HPMA) macro‐CTA was performed following RAFT‐PISA polymerization with PVK monomer in a methanol/water mixture in the presence of VA‐044 initiator at 50 °C. As seen in the initial molecular weight distribution (MWD) data in Tables S1–S3, all systems yielded well‐controlled polymers. Many well‐defined systems have been synthesized via RAFT‐PISA polymerization because of its ability to synthesize well‐controlled polymers with living characteristics and the ability to tolerate a wide range of monomer types.^[^ ^70^, ^71^ ^]^

**Figure 2:**
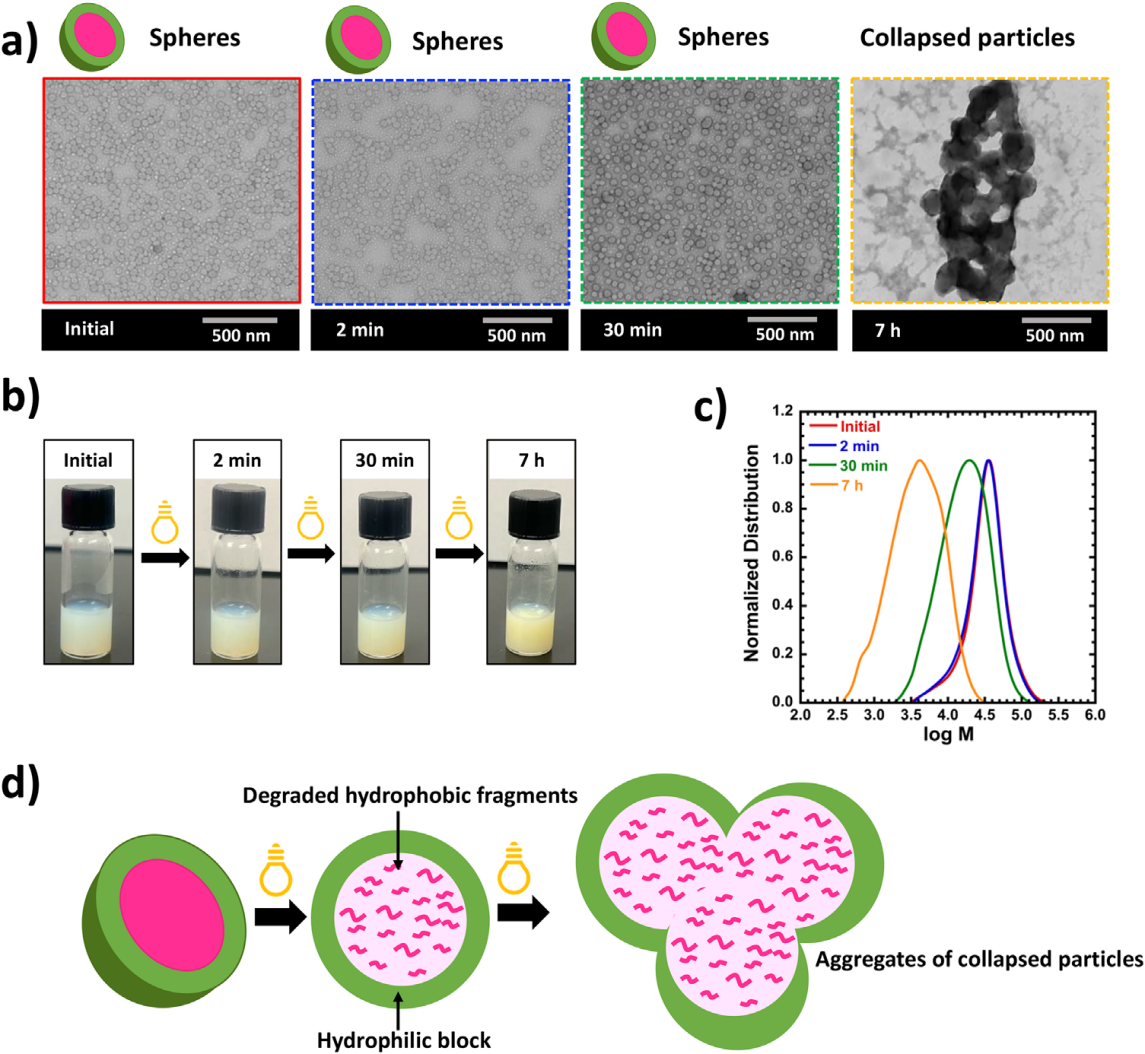
a) TEM images of sphere nanoparticles after RAFT‐PISA polymerization (at ∼100% monomer conversion) and photodegradation. Polymerization conditions: Molar ratio of PVK:poly(HPMA):VA‐044 = 400:1:0.2 in 50% MeOH as the solvent, at 10 w/w% solid concentration, and 50 °C. Degradation conditions: under 350 nm (Intensity = 2.5 ± 0.13 mW cm^−2^) at different time = 2 min, 30 min, and 7 h. b) Photographs of degraded and undegraded nanoparticle systems. c) Evolution of normalized molecular weight distributions at different degradation times. d) Schematic representation of the assembly of degraded PVK fragments in the hydrophobic core and the aggregation of collapsed particles.

**Figure 3:**
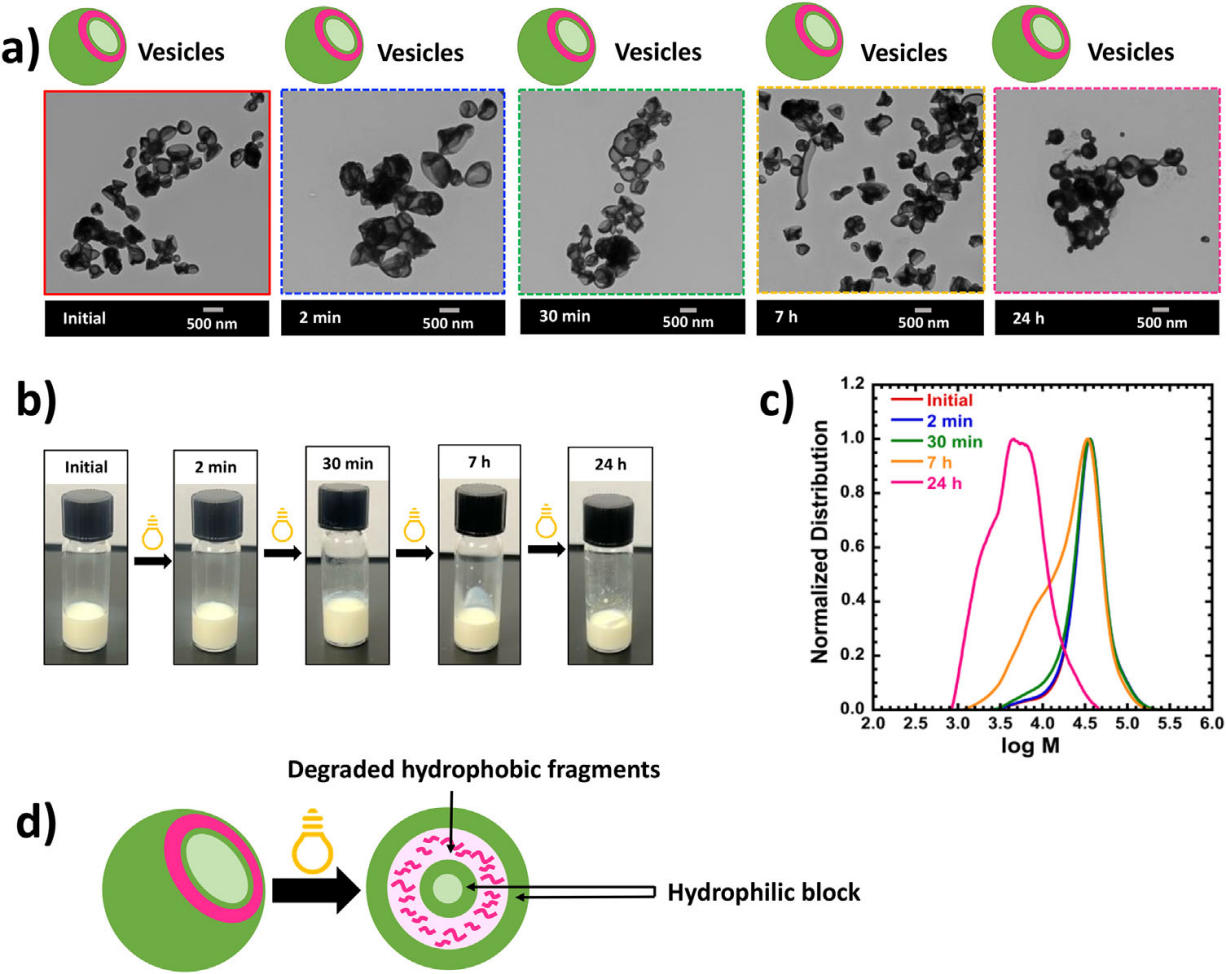
TEM images of vesicle nanoparticles after RAFT‐PISA polymerization (at ∼100% monomer conversion) and photodegradation. Polymerization conditions: Molar ratio of PVK:poly(HPMA):VA‐044 = 400:1:0.2 in 50% MeOH as the solvent, at 30 w/w% solid concentration, and 50 °C. Degradation conditions: under 350 nm (Intensity = 2.5 ± 0.13 mW cm^−2^) at different time = 2 min, 30 min, 7 h, and 24 h. b) Photographs of degraded and undegraded nanoparticle systems. c) Evolution of normalized molecular weight distributions at different degradation times. d) Schematic representation of the assembly of degraded PVK fragments in the hydrophobic region.

**Figure 4:**
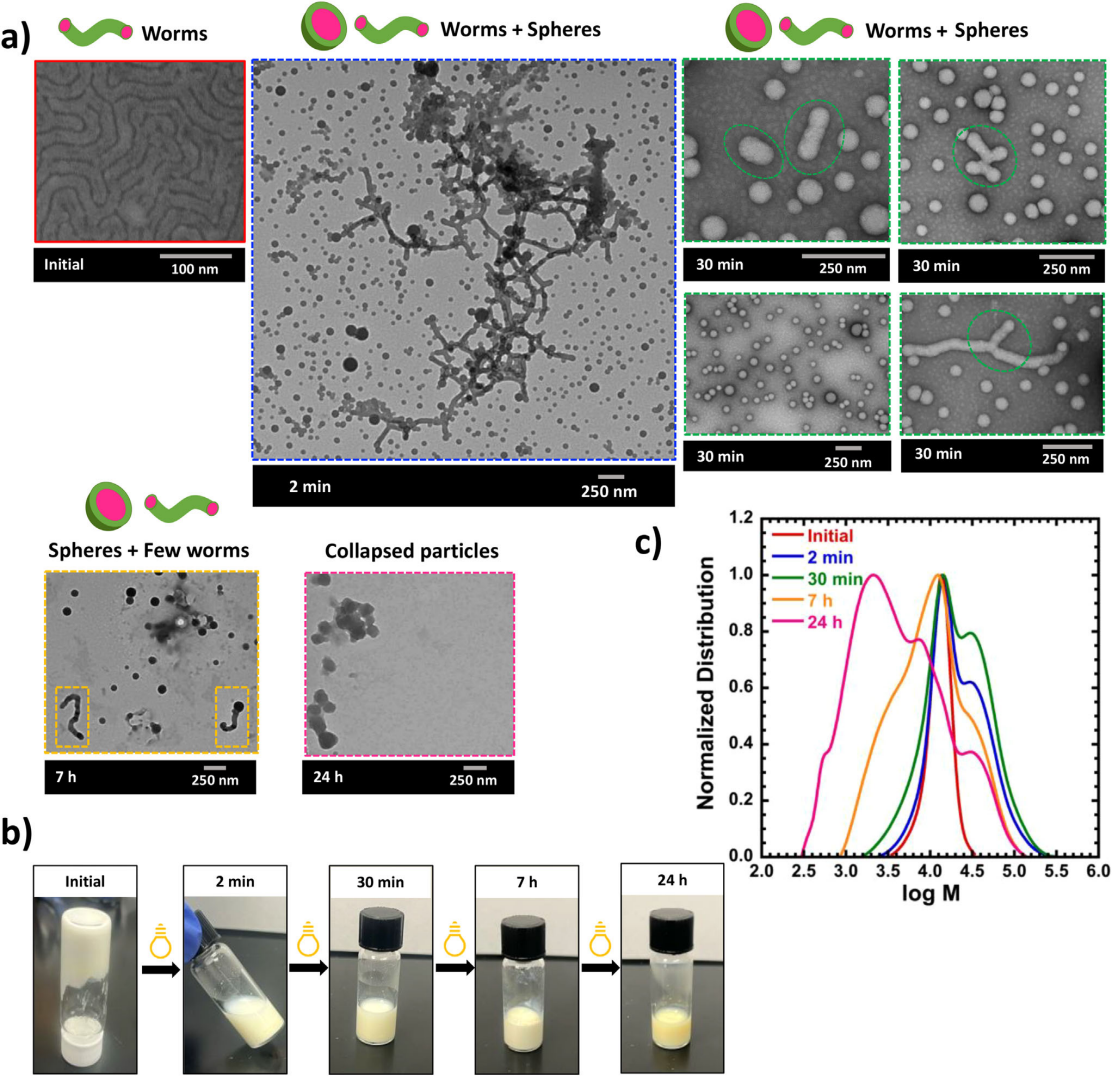
TEM images of worm nanoparticles after RAFT‐PISA polymerization (at ∼20% monomer conversion) and photodegradation. Polymerization conditions: Molar ratio of PVK:poly(HPMA):VA‐044 = 400:1:0.2 in 50% MeOH as the solvent, at 20 w/w% solid concentration, and 50 °C. Degradation conditions: under 350 nm (Intensity = 2.5 ± 0.13 mW cm^−2^) at different time = 2 min, 30 min, 7 h, and 24 h. b) Photographs of degraded and undegraded nanoparticle systems. c) Evolution of normalized molecular weight distributions at different degradation times.

First, a nanoparticle system was synthesized in the presence of 50% MeOH and 10 w/w% solid concentration. The system yielded polymer nanoparticles with *M_n _* = 26400 (*M_w_/M_n _* = 1.49) and conversion >95% after 20 h of polymerization time. As seen in Figure 2a, the spherical shape of particles in the initial sample (45 nm) was confirmed using transmission electron microscopy (TEM). The particle sizes of all morphologies were determined by analyzing TEM images in Image J software. After initial characterizations, particles were degraded under UV light at 350 nm (Intensity = 2.5 ± 0.13 mW/cm^2^) without any further dilutions. As illustrated in Figure 1c, the Norrish type II mechanism begins when UV light excites the carbonyl chromophore in ketones to the singlet excited state (*S*_1_). This excited state then undergoes intersystem crossing (ISC) to a excited triplet state (*T*_n_), which quickly relaxes to the lowest triplet state (*T*_1_) through internal conversion and vibrational relaxation. From the *T*_1_ state, the molecule performs a γ‐hydrogen abstraction, resulting in the formation of a 1,4‐biradical intermediate. This intermediate then undergoes photoinduced elimination (α−β bond dissociation). The reaction yields two distinct fragments, typically an alkene and a ketone, enabling light‐triggered cleavage in the polymer backbone. Our earlier detailed degradation kinetic work on vinyl ketone polymers indicated that the degradation of PVK polymers is fastest at 350 nm compared to 310 nm, and there is no degradation under visible light irradiation.^[^ ^58^ ^]^ However, spheres were subjected to 310 nm and 450 nm degradation to show their low or no impact compared to 350 nm (Figures S10 and S12). Under UV degradation conditions, the *M_n_* decreased gradually, and the initial MWD broadened due to random cleavage of the PVK polymer block under UV through the Norrish type II mechanism. This was shown in Figures 2, 3, 4 and S9a–h, and Tables S1–S3. Interestingly, the sphere shape and size of particles (∼45 nm, as determined by TEM) were unchanged in both 2‐min and 30‐min degraded samples in the system shown in Figure 2. Although the shape of particles was unchanged after 30 min of degradation, *M_n_* decreased by nearly 50% of the initial *M_n_*. The packing parameter (*P = v/al*) is a main concept in predicting the morphology of self‐assembled nanoparticles, where *v* is the volume of the hydrophobic block, *a* is the optimal area of the hydrophilic head group, and *l* is the length of the hydrophobic block. This parameter balances the ratio of hydrophilic to hydrophobic and determines the curvature and the final morphology.^[^ ^8^, ^72^ ^]^ Therefore, the unchanged shape of particles could be explained by considering the gathering of degraded PVK fragments in the hydrophobic core of spheres, as illustrated in Figure 2d. However, after 7 h of photodegradation, the system included aggregates of collapsed particles. This could be due to loss of colloidal stability. The reduced colloidal stability could be caused by the photodegraded poly(HPMA)‐b‐poly(PVK) copolymer with the shorter PVK segment being more soluble in the continuous phase, thereby reducing steric stabilization of the nanoparticles. A similar degradation pattern was observed in all systems with initial sphere nanoparticles (Figures S2, S3, and S5). TEM images shown in Figure S11 present the morphology of sphere particles degraded at 310 nm. Compared to the 350 nm system, at the 310 nm, particles retained a visible spherical morphology with some distortion even after 7 h of degradation, whereas complete collapse of the particles was observed in the 350 nm system after the same degradation period. This occurs due to the lower degradation efficiency of PVK chains at 310 nm^[^ ^58^ ^]^ (Table S8) and relatively higher scattering effect at 310 nm compared to 350 nm,^[^ ^73^ ^]^ resulting in less pronounced changes under light.

As the second system, the degradation behavior of vesicle‐shaped nanoparticles was explored. First, vesicle nanoparticles were synthesized by following RAFT‐PISA polymerization for the system with 50% MeOH and 30 w/w% solid concentration. After 24 h of reaction time, the system reached >95% conversion and resulted in particles with *M_n _* = 28500 (*M_w_/M_n _* = 1.38). Initial vesicles were ∼365 nm (as determined by TEM) in size and were larger than both worm and sphere particles. Surprisingly, vesicle‐shaped particles remained unchanged even after 24 h of degradation. This behavior could also be seen in all other vesicle nanoparticle systems made under different conditions, as seen in Figures S4, S7, and S8 (e.g., 60% MeOH systems at 20 w/w% solid concentration and 60% MeOH and 75% MeOH systems at 30 w/w% solid concentration). Moreover, the size data shows that the size of particles in initial to degraded samples were varied in the same range without showing a significant difference (2‐min degraded = 460 nm, 30‐min degraded = 390 nm, 7‐h degraded = 460 nm, and 24‐h degraded = 400 nm, as determined by TEM) even though the reduction of *M_n_* is significant especially after 30 min or 7 h of degradation. For instance, the %*M_n_* reduction of the 7‐h and 24‐h degraded samples with respect to the initial *M_n_* is ∼50% and ∼87% in the system discussed under Figure 3. The unchanging behavior of the shape and size of vesicles at both the initial and later stages of degradation could be due to all the degraded PVK fragments assembling in the hydrophobic region of the vesicles, maintaining the hydrophobic volume. This causes retention of the vesicle shape because the morphology of a nanoparticle system is dictated by the volume ratio of hydrophilic and hydrophobic blocks.^[^ ^7^, ^72^, ^74^, ^75^ ^]^ In addition to that, as seen in Figures 2b and 3b, photographic images of the sphere and vesicle nanoparticles, upon degradation, show a slight change in the turbidity of the solution and a very minor degree of sedimentation over time.

The third nanoparticle system was prepared in 50% MeOH and in the presence of 20 w/w% solid concentration, which lies between the 10% that generated spheres and the 30% that generated vesicles. The system yielded a gel‐like material with *M_n _* = 11900 (*M_w_/M_n _* = 1.13) and 20% conversion in 15 min of polymerization time. The high viscosity suggested entanglements of worm particles. The initial image of Figure 4a confirms worm particles with a 19 nm (width) size (as determined by TEM). Unfortunately, isolating worms from unreacted PVK monomers was challenging without disturbing the assembly. Furthermore, the previous studies of photopolymerization of PVK monomer have shown that although UV is capable of starting chain growth of PVK monomer, after some point, the coinciding degradation process of the resulting PVK polymer becomes prominent and eventually degrades all fragments.^[^ ^76^ ^]^ Therefore, the degradation of worm particles was under the impact of both polymerization and degradation until 7 h, because after 7 h, all unreacted monomers were consumed. The initial system had *M_n _* = 11900 (*M_w_/M_n _* = 1.13) and 20% monomer conversion. In contrast, UV irradiation: at 2 min gave *M_n _* = 16900 (*M_w_/M_n _* = 1.75) and 24% monomer conversion; at 30 min gave *M_n _* = 14500 (*M_w_/M_n _* = 2.06) and 46% monomer conversion; and finally, at 7 h gave *M_n _* = 6000 (*M_w_/M_n _* = 2.57) and >95% monomer conversion. The MWD in Figure 4c shows shoulder peaks at both low and high molecular weight sides of the initial peak. The high molecular weight shoulders were not present in the systems that started as either spheres or vesicles. This high molecular weight peak is either due to chain extension of the poly(HPMA)‐b‐poly(PVK) with unreacted PVK or radical coupling. The radical coupling products could be more pronounced in the worm system with unreacted PVK, due to more efficient radical generation from the unreacted PVK monomer, whereas this unreacted PVK was absent in the sphere and vesicle morphologies.

The changes in molecular weight were reflected in the viscosity and morphological properties of the PISA system. After 2 min of degradation, the initial high viscosity worm sample turned into a relatively low viscosity sample, suggesting a quick and drastic morphological transition, as seen in Figure 4b photographic images. TEM analysis further revealed that the system included a mixture of spheres with a 48 nm diameter and long, branched worm particles with a 47 nm width (as determined by TEM). Interestingly, inspecting the MWD data in Figure 4c suggests the formation of a shoulder peak with high molecular weight. This could be due to the polymerization of some chains forming higher molecular weight polymers via chain extension with unreacted PVK, coupled with the formation of dead chains through degradation. Additionally, the radical generation from the unreacted PVK monomer could also lead to higher radical fluxes that also promote radical coupling, compared to systems with only polymer present. The sample degraded for 30 min contained more sphere particles and some shorter worm fragments. Especially, as highlighted in green circles (TEM images after 30 min of degradation), those degraded worm fragments appeared as shorter fragments made with the fusion of spheres or the center of a 3‐ or 4‐way branched worm.

The consumption of unreacted monomers and competition of polymerization and degradation could happen through the diffusion of hydrophobic PVK monomers to the hydrophobic core of nanoparticles and consumption in the hydrophobic core. Furthermore, based on the size comparison, the width of degraded worm fragments or the diameter of spheres after 2 min of degradation is higher than the width of the initial worms. This contributes to the initial hypothesis, which is the solubilization of unreacted PVK monomer into the hydrophobic core and then its utilization in the hydrophobic core.

**Figure 5:**
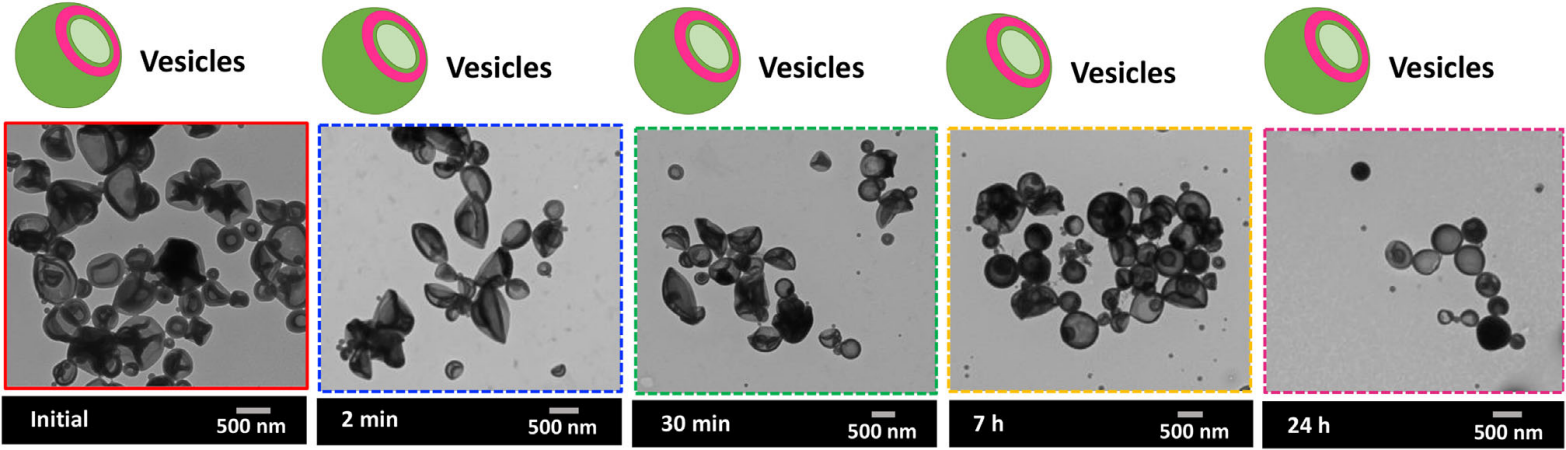
TEM images of vesicle nanoparticles after RAFT‐PISA polymerization (at ∼100% monomer conversion) and photodegradation. Polymerization conditions: Molar ratio of PVK:poly(HPMA):VA‐044 = 400:1:0.2 in 50% MeOH as the solvent, at 20 w/w% solid concentration, and 50 °C. Degradation conditions: under 350 nm (Intensity = 2.5 ± 0.13 mW/cm^2^) at different time = 2 min, 30 min, 7 h, and 24 h.

Additionally, the 50% MeOH system with 20 w/w% solid concentration (which yields worm particles at 20% conversion) was kept for 24 h until it reached >95% conversion. The reaction medium appeared as a milky color and less viscous solution similar to the initial photographic image of vesicle solution shown in Figure 3b, generated at 30 w/w% solid concentration. Despite forming worms near 20% PVK conversion, the same system yielded >95% PVK conversion after 24 h, giving vesicle nanoparticles with *M_n _* = 33300 (*M_w_/M_n _* = 1.47) and 510 nm size compared to worm particles with 19 nm width (as determined by TEM). Similar to other vesicle systems, vesicle shape was unchanged with minimum size variation (2‐min degraded = 460 nm, 30‐min degraded = 525 nm, 7‐h degraded = 515 nm, and 24‐h degraded = 445 nm, as determined by TEM) even after 24 h of degradation, although *M_n_* decreased with degradation as seen in Table S2 and Figure S9C. When comparing the worm system shown in Figure 4 and the vesicle system shown in Figure 5 (both systems were made under the same 50% MeOH and 20 w/w% solid concentration), they have demonstrated totally different photodegradation behavior. The worm system showed a dramatic morphological change in 2 min of degradation, while the vesicle system maintained its morphology even after 24 h of degradation. Furthermore, particle size and *M_n_* increased during the degradation until all unreacted monomers in the worm system were completely consumed. This difference in photodegradation could happen because of the lower stability of worm particles under light and due to the presence of unreacted monomers in the worm system.

**Figure 6:**
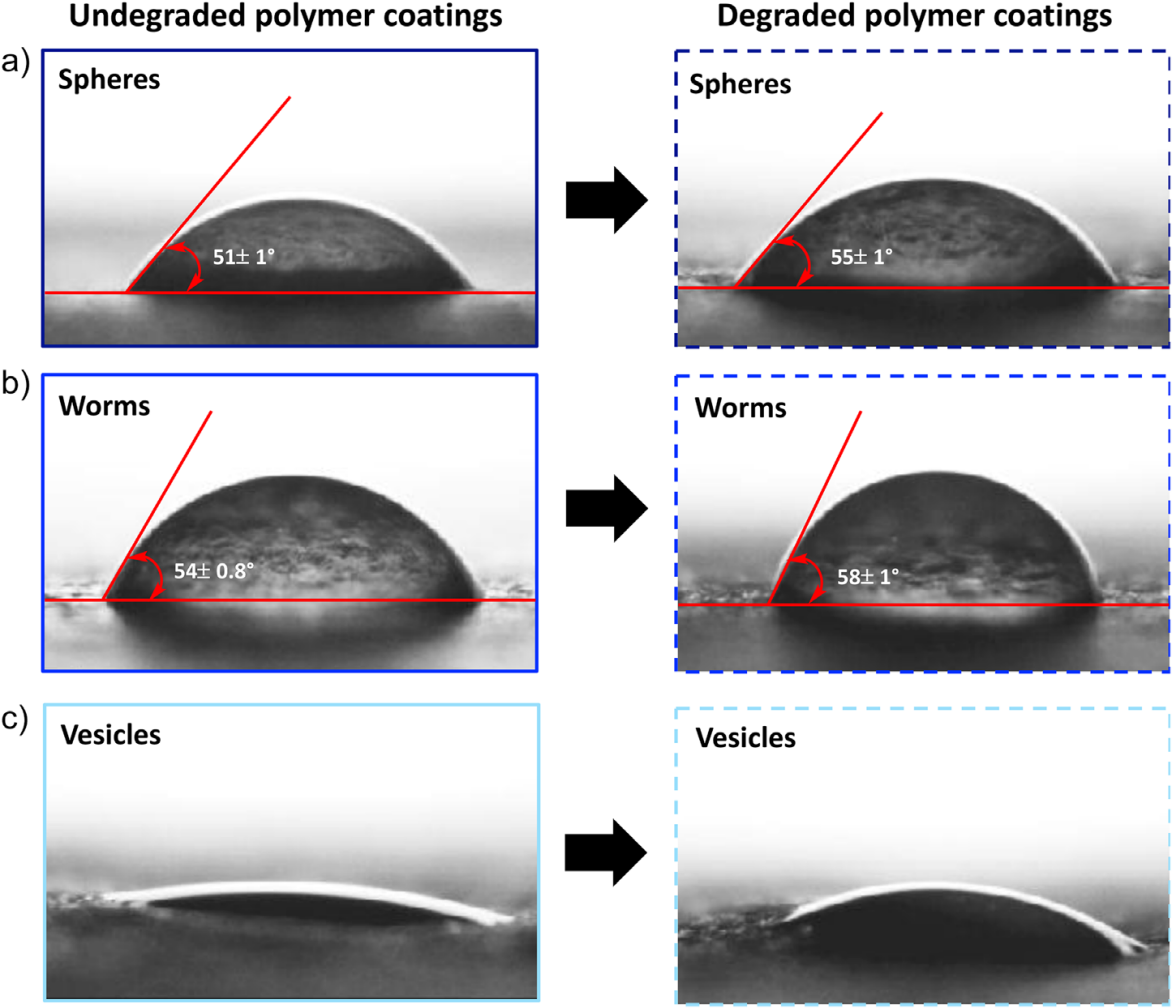
Contact angle measurements of water on polymer nanoparticle coatings applied to a paper surface. a) Water drop on undegraded and degraded sphere coatings applied to a paper surface. b) Water drop on undegraded and degraded worm coatings applied to a paper surface. c) Water drop on undegraded and degraded vesicle coatings applied to a paper surface.

To evaluate if changes in particle morphology impact the final material properties, the surface properties of polymer films deposited on paper substrates were studied. The surface properties of polymer‐coated paper made with undegraded and degraded sphere, worm, and vesicle nanoparticles on a paper surface were determined by measuring the contact angle (*θ*) of a water drop. Figures 6 and S13 highlight the difference between the contact angle of the water drop on different nanoparticle coatings. The unmodified paper had a contact angle of 65 ± 3° with water. The contact angles of undegraded and degraded sphere coatings were 51 ± 1° and 55 ± 1°, respectively. While the contact angles of undegraded and degraded worm coatings were measured as 54 ± 0.8° and 58 ± 1°, respectively. In all cases, the unmodified paper, undegraded and degraded sphere, and worm coatings had contact angles below 90°, suggesting a hydrophilic surface. Importantly, the contact angle decreases upon polymer modification, suggesting the hydrophilic groups in the complex polymer facilitate the high wettability of the surface compared to the unmodified paper. Further, a statistically significant (P‐value < 1%) increment of the contact angle was observed for degraded nanoparticle coatings compared to undegraded nanoparticle coatings, showing a relatively higher hydrophobic nature of the surface coated with degraded nanoparticles. This measurable increase in contact angle could result from the presence of some hydrophobic fragments in the solution after the degradation of the hydrophobic PVK block. Interestingly, both undegraded and degraded vesicle‐coated surfaces showed different behavior upon the addition of a water drop. The added drop quickly spread out on the surface, showing high hydrophilicity and high wettability compared to all undegraded and degraded spheres and worm‐coated surfaces (Figure 6c and supplemental movies). This high hydrophilicity may be attributed to the lamella‐like arrangement of vesicle‐derived nanoparticles on the paper after drying.

**Figure 7:**
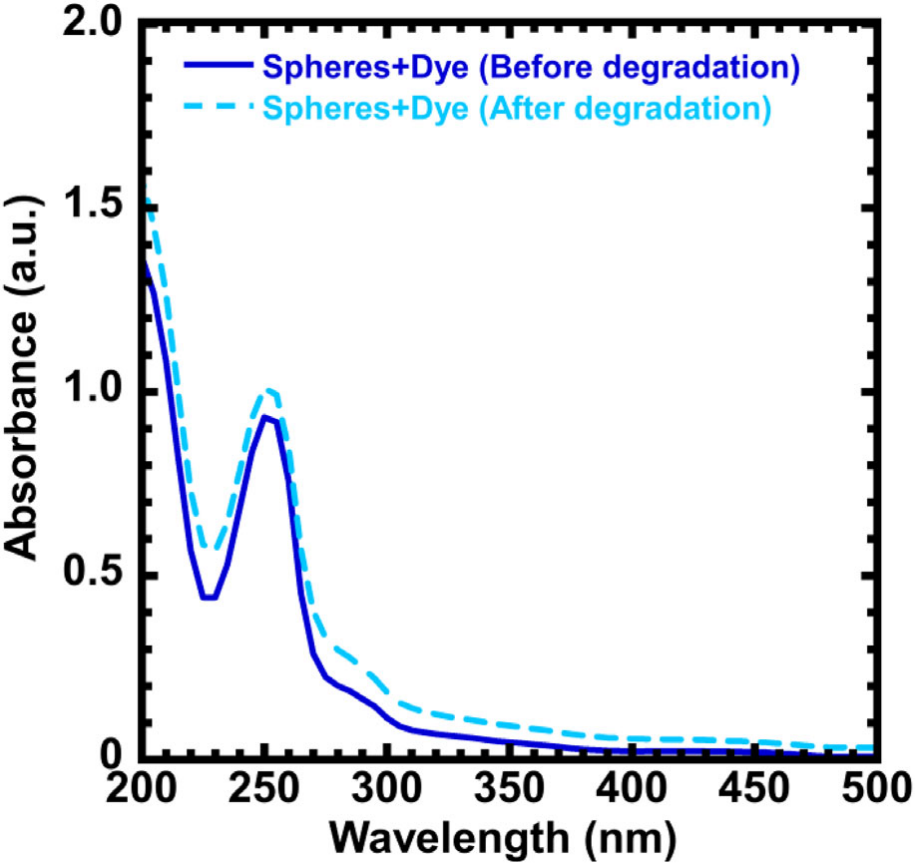
Absorbance spectra of sphere nanoparticles encapsulated with 9‐fluorenone dye molecules before and after degradation. Dye encapsulation: 500 µL of sphere nanoparticles made with 60% MeOH was mixed with 50 µL of 9‐fluorenone dye. (0.125 mgµL^−1^, dissolved in methanol). Degradation conditions: under 350 nm (Intensity = 2.5 ± 0.13 mW cm^−2^) for 24 h.

The ability of photodegradable PVK nanoparticles to release small, encapsulated molecules upon degradation was explored. 9‐Fluorenone was selected as the model encapsulating agent to demonstrate the concept because of its hydrophobic and UV‐active nature.^[^ ^77^ ^]^ The sphere nanoparticles made with 60% MeOH were encapsulated with 9‐fluorenone (50 µL from 0.125 mgµL^−1^). After encapsulation, particles were degraded for 24 h under 350 nm to trigger the release of the dye. The dye release was characterized by measuring the UV absorbance of the dye. Figure S14 presents the UV spectrum of 9‐fluorenone dye, and Figure S15 presents the comparison of absorbance between samples containing degraded spheres, undegraded spheres, and undegraded spheres loaded with the dye. A reduction in the absorbance of the peak around 250 nm is observed in the degraded sphere sample compared to the undegraded sphere sample (Figure S15), which may correspond to the disruption of phenyl rings in the degradable PVK block. The sample with the undegraded spheres loaded with the dye shows an increased absorbance. As shown in Figure 7, absorbance increases after degradation, confirming dye release due to the degradation of the PVK block and highlighting the ability of PVK nanoparticles to release encapsulated molecules upon photodegradation. Furthermore, TEM images of sphere nanoparticles before and after dye encapsulation are presented in Figure S16. While the primary spherical morphology remained unchanged following dye encapsulation, the average particle size increased from approximately 33 nm to about 46 nm (as determined by TEM), indicating the particle size change as a result of the dye encapsulation.

## Conclusion

Photo responsive phenyl vinyl ketone polymer nanoparticles with different morphologies, including sphere, worm, and vesicle were synthesized via the PISA technique, varying solvent composition, solid concentration, and polymerization time. Photo degradability of nanoparticles was explored under UV light to see the impact of degradation of the PVK hydrophobic block on morphology. Sphere, worm, and vesicle particles behaved differently under light, and their changes were evaluated by determining the *M_n_* (GPC), morphology (TEM), and size with respect to the degradation time. Sphere particles maintained their shape and size until 7 h of degradation under light, with ultimate collapse of the nanoparticles to poorly defined aggregates, while the morphology of vesicle particles was unchanged even after 24 h of degradation. This could be explained by the degraded PVK fragments remaining in the hydrophobic region to maintain the volume ratio of hydrophilic to hydrophobic segments. However, the degradation of worm particles was under the impact of both the concurrent degradation of the polymer and the polymerization of unreacted monomers in the system. Worms showed rapid morphology change within 2 min of degradation, reversing their morphology into spheres, despite minimal change in monomer conversion over this timeframe. The consumption of unreacted monomers in the system could be explained by hypothesizing the solubilization of PVK monomers into the hydrophobic core. The PISA‐generated amphiphilic polymers were subsequently deposited to modify the surface of paper. All polymer‐coated surfaces were more hydrophilic than the unmodified paper, with worms leading to the least hydrophilic surface, followed by spheres, and vesicles leading to a very hydrophilic surface with rapid wetting. Furthermore, in the case of surfaces generated from sphere or worm morphologies, the hydrophilicity decreased after photodegradation of the vinyl ketone polymers. This indicates that both the primary morphology and the extent of degradation impact surface coatings. Finally, the ability of PVK nanoparticles to release encapsulated molecules upon degradation was successfully confirmed. This work explored the degradation behavior of different shape photo responsive PVK nanoparticles under light, which could ultimately lead to potential new applications where the degradability under light is an added value compared to traditional nondegrading polymer nanoparticles.

## Conflict of Interests

The authors declare no conflict of interest.

## Supporting information



Supporting Information

Supporting Information

Supporting Information

## Data Availability

The data that support the findings of this study are openly available in [Miami University Scholarly Commons] at [http://hdl.handle.net/2374.MIA/7031], reference number [7031].
