# Supplementary material for: Changing Self‐Assembly Through Degradation: Phenyl Vinyl Ketone Polymer Nanoparticles Under Light
Source: Angew Chem Int Ed Engl. 2025 Sep 8;64(44):e202514545. doi: 10.1002/anie.202514545 (PMC12559474; doi:10.1002/anie.202514545)
Supplement: Supplementary file 1 — Supporting Information [file ANIE-64-e202514545-s002.docx]

**Supporting Information**

**Changing Self-Assembly through Degradation: Phenyl Vinyl Ketone Polymer Nanoparticles under Light.**

M. A. Sachini N. Weerasinghe,^a^ Parker Anthony McBeth,^a^ Michelle C. Mancini,^a^ Dominik Konkolewicz ^a*^

^a^  Department of Chemistry and Biochemistry, Miami University, 651 E High St, Oxford, 45056, OH, USA

*Correspondence: [d.konkolewicz@miamiOH.edu](mailto:d.konkolewicz@miamiOH.edu)

**Methods**

***Characterizations***

*Nuclear Magnetic Resonance (NMR) Spectroscopy*

^1^H NMR spectra were obtained on a Bruker 400 MHz spectrometer. Monomer conversions were determined via proton (^1^H) NMR spectroscopy. Data are reported in ppm using DMSO as the solvent, unless otherwise specified.

*Size exclusion Chromatography (SEC)*

All size exclusion chromatography (SEC) was performed using an Agilent SEC system equipped with an autosampler, an Agilent 1260 isocratic pump, 1 × Agilent Mixed-B guard and 2 × Agilent Mixed-B analytical columns, and an Agilent 1260 refractive index (RI) detector. The eluent, dimethylformamide (DMF) was at 50 °C with a flow rate of 1 mL/min. The system was calibrated with poly(methyl methacrylate) standards having molecular weights in the range of 617,500 to 1,010. All samples were filtered through a 200 nm PTFE filter before injection. Theoretical molecular weights were calculated as *M_n, Theory_* =M_Monomer_ × ([Monomer]_o_ /[CTA]_o_) × Monomer Conversion, where M_Monomer_ is the molecular weight of monomer, [Monomer]_o_ is the initial concentration of monomer, and [CTA]_o_ is the initial concentration of the CTA.

*Transmission electron microscopy (TEM)*

All samples were diluted 300-fold with methanol before TEM imaging. TEM samples were prepared by first placing a carbon-coated copper grid (carbon-coated side on the drop) on a drop of sample for 60 s, followed by blotting of excess and air drying. Then again, the grid with the sample was placed on a drop of staining, which was a commercially purchased UranyLess staining for 60 s. The excess stains were blotted, and the grid was air dried. The prepared samples were stored under ambient conditions until imaging. The stained samples were imaged using a JeoL 1200 transmission electron microscope (**Electron Sources:** Tungsten Hairpin & LaB6, **Accelerating Voltages:** 40 to 120 kV).

*Dynamic Light Scattering (DLS)*

All samples were diluted 300-fold with methanol before the analysis. The samples were analyzed using a Malvern Zetasizer-nano series instrument at 25 °C. The sample refractive index (RI) was set at 1.51 for PVK. The dispersant (methanol) viscosity and RI were set to 0.5476 cP and 1.326, respectively.

*Contact Angle Measurement*

Sphere, worm, and vesicle nanoparticle samples were coated on paper (copy paper). Coatings were dried in the oven at 40 °C for 1 h. The contact angle of a water drop on polymer coatings was measured using a PGX+ Contact Angle Goniometer. A similar procedure was followed for surfaces coated with degraded sphere, worm, and vesicle nanoparticle samples. The degraded nanoparticle samples were obtained by degrading samples under 350 nm (Intensity=2.5 ± 0.13 mW/cm^2^) for 24 h. Differences between the water contact angle on polymer-modified paper before and after UV degradation were evaluated using a Student’s t-test of means.

*UV Analysis*

UV absorbance spectra of samples with spheres, degraded spheres, 9-fluorenone, undegraded (spheres + 9-fluorenone), and degraded (spheres + 9-fluorenone) were measured using an Agilent Cary 60 UV-Vis spectrometer. The degraded samples were obtained by degrading samples under 350 nm (Intensity=2.5 ± 0.13 mW/cm^2^) for 24 h.

***Materials***

All materials, including 2-hydroxypropyl methacrylamide (HPMA) and 2,2′-Azobis[2-(2-imidazolin-2-yl)propane]dihydrochloride (VA-044), and 9-fluorenone were purchased from commercially available sources and used without purification unless otherwise specified. The RAFT agents (S-2-cyanoisopropyl S′-ethyl trithiocarbonate (CPETC)^[1]^ and 1-phenylprop-2-en-1-one (PVK)^[2]^ were synthesized as in the literature procedure.

***Synthesis***

*Typical procedure for RAFT polymerization of 2-hydroxypropyl methacrylamide-Poly(HPMA)*

Poly(HPMA) was synthesized using a modified literature procedure.^[3]^ The molar ratio HPMA:CPETC:VA-044=25:1:0.2 and the mass ratio of HPMA:methanol=1:2 were used for the polymerization. A 10 mL round-bottom flask was equipped with a Teflon stir bar, and HPMA (2.0 g, 14.0x10^-3^ mol), CPETC (114.7 mg, 5.6x10^-4^ mol), VA-044 (36.1 mg, 1.1x10^-4^ mol), and methanol (4.0 g) were added. The reaction mixture was deoxygenated by bubbling N_2_ for 25 min. The polymerization reaction was carried out in an oil bath at 50 ºC for 48 h. The sample was analyzed using ^1^H NMR (DMSO) and GPC (DMF). The dried polymer was obtained after precipitation in ethyl ether and vacuum drying.

*Typical procedure for RAFT-PISA polymerization to prepare nanoparticles*

The mole ratio of poly(HPMA)-CTA:PVK:VA-044=1:400:0.2 was measured in a 10 mL vial with a Teflon stir bar. The molar ratio of poly(HPMA)-CTA:PVK:VA-044 was kept constant in all systems. The solvent system made with methanol and water ((MeOH:Water=50:50)=50% MeOH), ((MeOH:Water=60:40)=60% MeOH), and ((MeOH:Water=75:25)=75%MeOH)) was used as the solvent, and the added amount was determined based on the total solid concentration. (10%, 20%, and 30% solid concentrations). The mixtures were sealed with a septum and deoxygenated by bubbling N_2_ for 25 min. The polymerization reaction was carried out in an oil bath at 50 ºC. The polymer sample was analyzed using ^1^H NMR (DMSO), GPC (DMF), TEM, and DLS.

*Typical degradation procedure*

Nanoparticle sample (250 μL) was added to a glass tube (length=5 cm, outer diameter=5 mm) without any dilutions, and the system was sealed with a cap. Degradation was carried out under UV light. (Description of the UV reactor – Consists of 16 UV bulbs (λ=350 nm, total intensity=2.5± 0.13 mW/cm^2^, inner diameter of the reactor=24 cm, and depth of the reactor=38 cm) at different time intervals. (2 min, 30 min, 7 h, and 24 h). The degraded samples were analyzed using GPC (DMF), TEM, and DLS.

The typical degradation procedure was followed under visible (blue) light irradiation. (Description of the blue light reactor – Consists of blue LED strips (λ=450 nm, total intensity=1.9±0.20 mW/cm^2^, diameter of the reactor=15 cm, and depth of the reactor=14 cm).

*Encapsulation procedure*

500 μL of sphere sample made with 50% MeOH and 10% solid concentration, mixed with 50 μL of 9-fluorenone (0.125 mgμL^-1^, in methanol) and stirred for 30 minutes. The degraded samples were obtained by following the degradation under 350 nm (Intensity=2.5 ± 0.13 mW/cm^2^) for 24 h.

**Supplemental Data**

**
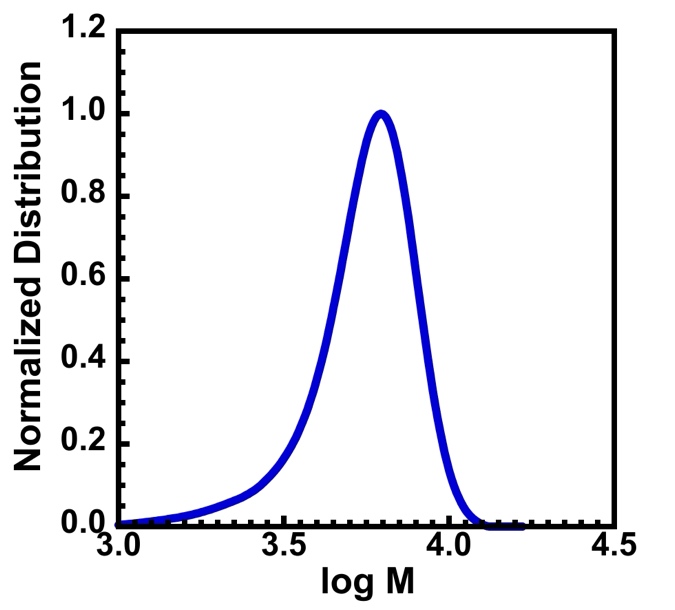
**

**Figure S1:** Molecular weight distribution of poly(HPMA) block.

**Figure S2:** TEM images of sphere nanoparticles after RAFT-PISA polymerization (at ~100% monomer conversion) and photodegradation. Polymerization conditions: Molar ratio of PVK:poly(HPMA):VA-044=400:1:0.2 in 60% MeOH as the solvent, at 10 w/w% solid concentration, and 50 °C. Degradation conditions: under 350 nm (Intensity=2.5 ± 0.13 mW/cm^2^) at different time=2 min, 30 min, and 7 h.

**Figure S3:** TEM images of sphere nanoparticles after RAFT-PISA polymerization (at ~100% monomer conversion) and photodegradation. Polymerization conditions: Molar ratio of PVK:poly(HPMA):VA-044=400:1:0.2 in 75% MeOH as the solvent, at 10 w/w% solid concentration, and 50 °C. Degradation conditions: under 350 nm (Intensity=2.5 ± 0.13 mW/cm^2^) at different time=2 min, 30 min, and 7 h.

**Figure S4:** TEM images of vesicle nanoparticles after RAFT-PISA polymerization (at ~100% monomer conversion) and photodegradation. Polymerization conditions: Molar ratio of PVK:poly(HPMA):VA-044=400:1:0.2 in 60% MeOH as the solvent, at 20 w/w% solid concentration, and 50 °C. Degradation conditions: under 350 nm (Intensity=2.5 ± 0.13 mW/cm^2^) at different time=2 min, 30 min, 7 h, and 24 h.

**Figure S5:** TEM images of sphere nanoparticles after RAFT-PISA polymerization (at ~100% monomer conversion) and photodegradation. Polymerization conditions: Molar ratio of PVK:poly(HPMA):VA-044=400:1:0.2 in 75% MeOH as the solvent, at 20 w/w% solid concentration, and 50 °C. Degradation conditions: under 350 nm (Intensity=2.5 ± 0.13 mW/cm^2^) at different time=2 min, 30 min, 7 h, and 24 h.

**Figure S6:** TEM images of worm nanoparticles after RAFT-PISA polymerization (at ~16% monomer conversion) and photodegradation. Polymerization conditions: Molar ratio of PVK:poly(HPMA):VA-044=400:1:0.2 in 50% MeOH as the solvent, at 30 w/w% solid concentration, and 50 °C. Degradation conditions: under 350 nm (Intensity=2.5 ± 0.13 mW/cm^2^) at different time=2 min, 30 min, and 7 h.

**Figure S7:** TEM images of vesicle nanoparticles after RAFT-PISA polymerization (at ~100% monomer conversion) and photodegradation. Polymerization conditions: Molar ratio of PVK:poly(HPMA):VA-044=400:1:0.2 in 60% MeOH as the solvent, at 30 w/w% solid concentration, and 50 °C. Degradation conditions: under 350 nm (Intensity=2.5 ± 0.13 mW/cm^2^) at different time=2 min, 30 min, 7 h, and 24 h.

**Figure S8:** TEM images of vesicle nanoparticles after RAFT-PISA polymerization (at ~100% monomer conversion) and photodegradation. Polymerization conditions: Molar ratio of PVK:poly(HPMA):VA-044=400:1:0.2 in 75% MeOH as the solvent, at 30 w/w% solid concentration, and 50 °C. Degradation conditions: under 350 nm (Intensity=2.5 ± 0.13 mW/cm^2^) at different time=2 min, 30 min, 7 h, and 24 h.

**Figure S9:** Evolution of normalized molecular weight distributions at different degradation times. (Initial, 2 min, 30 min, 7 h, and 24 h). (a) Nanoparticle system made with 60% MeOH as the solvent and at 10 w/w% solid concentration. (b) Nanoparticle system made with 75% MeOH as the solvent and at 10 w/w% solid concentrations. (c) Nanoparticle system made with 50% MeOH as the solvent and at 20 w/w% solid concentrations (at ~100%). (d) Nanoparticle system made with 60% MeOH as the solvent and at 20 w/w% solid concentration. (e) Nanoparticle system made with 75% MeOH as the solvent and at 20 w/w% solid concentration. (f) Nanoparticle system made with 50% MeOH as the solvent and at 30 w/w% solid concentration (at ~16%). (g) Nanoparticle system made with 60% MeOH as the solvent and at 30 w/w% solid concentration. (h) Nanoparticle system made with 75% MeOH as the solvent and at 30 w/w% solid concentration.

**Table S1:** Conversion%, *M_n,_* and *M_w_/M_n_* of initial nanoparticle systems made at 10% solid concentration and *M_n_* and *M_w_/M_n_* of degraded nanoparticles under light.

| **System** | **Conv.** | **Initial** | | **2 min** | | **30 min** | | **7 h** | | **24 h** | |
| --- | --- | --- | --- | --- | --- | --- | --- | --- | --- | --- | --- |
|  |  | ***M_n_*** | ***M_w_/M_n_*** | ***M_n_*** | ***M_w_/M_n_*** | ***M_n_*** | ***M_w_/M_n_*** | ***M_n_*** | ***M_w_/M_n_*** | ***M_n_*** | ***M_w_/M_n_*** |
| 50% MeOH | >95% | 26400 | 1.49 | 25400 | 1.47 | 12500 | 1.69 | 2600 | 1.90 | 1900 | 1.82 |
| 60% MeOH | >95% | 30000 | 1.50 | 25200 | 1.51 | 7900 | 1.66 | 2600 | 1.91 | 1900 | 1.91 |
| 75% MeOH | >95% | 29500 | 1.47 | 21700 | 1.60 | 8300 | 1.72 | 2300 | 2.00 | 2000 | 1.90 |

**Table S2:** Conversion%, *M_n,_* and *M_w_/M_n_* of initial nanoparticle systems made at 20% solid concentration, and *M_n_* and *M_w_/M_n_* of degraded nanoparticles under light.

| **System** | **Conv.** | **Initial** | | **2 min** | | **30 min** | | **7 h** | | **24 h** | |
| --- | --- | --- | --- | --- | --- | --- | --- | --- | --- | --- | --- |
|  |  | ***M_n_*** | ***M_w_/M_n_*** | ***M_n_*** | ***M_w_/M_n_*** | ***M_n_*** | ***M_w_/M_n_*** | ***M_n_*** | ***M_w_/M_n_*** | ***M_n_*** | ***M_w_/M_n_*** |
| 50% MeOH | 20% | 11900 | 1.13 | 16900 | 1.75 | 14500 | 2.06 | 6000 | 2.57 | 2200 | 4.32 |
| 50% MeOH | >95% | 33300 | 1.47 | 30800 | 1.57 | 22600 | 1.77 | 8900 | 2.44 | 4400 | 1.76 |
| 60% MeOH | >95% | 28400 | 1.38 | 26900 | 1.45 | 20100 | 1.59 | 8700 | 2.21 | 4100 | 2.05 |
| 75% MeOH | >95% | 30500 | 1.44 | 25600 | 1.60 | 12600 | 1.80 | 4500 | 1.65 | 2500 | 2.06 |

**Table S3:** Conversion%, *M_n,_* and *M_w_/M_n_* of initial nanoparticle systems made at 30% solid concentration, and *M_n_* and *M_w_/M_n_* of degraded nanoparticles under light.

| **System** | **Conv.** | **Initial** | | **2 min** | | **30 min** | | **7 h** | | **24 h** | |
| --- | --- | --- | --- | --- | --- | --- | --- | --- | --- | --- | --- |
|  |  | ***M_n_*** | ***M_w_/M_n_*** | ***M_n_*** | ***M_w_/M_n_*** | ***M_n_*** | ***M_w_/M_n_*** | ***M_n_*** | ***M_w_/M_n_*** | ***M_n_*** | ***M_w_/M_n_*** |
| 50% MeOH | 16% | 9300 | 1.14 | 9700 | 1.16 | 11400 | 1.50 | 5500 | 1.52 | 3400 | 1.95 |
| 50% MeOH | >95% | 28500 | 1.38 | 28300 | 1.38 | 25400 | 1.49 | 13600 | 1.99 | 3700 | 1.74 |
| 60% MeOH | >95% | 28600 | 1.37 | 27100 | 1.40 | 20200 | 1.61 | 4300 | 2.95 | 2900 | 3.13 |
| 75% MeOH | >95% | 27700 | 1.34 | 27300 | 1.38 | 17900 | 1.68 | 4600 | 2.35 | 2500 | 2.22 |

**Table S4:** Size (volume) and %distribution of initial nanoparticle systems made at 10% solid concentration and their degraded nanoparticles under light.

(a) (b) (c)

| 50% MeOH | Size/nm | % |  | 60% MeOH | Size/nm | % |  | 75% MeOH | Size/nm | % |
| --- | --- | --- | --- | --- | --- | --- | --- | --- | --- | --- |
| **Initial**  (>97% monomer conversion) | 22 | 99.6 |  | **Initial**  (>99% monomer conversion) | 21 | 99.6 |  | **Initial**  (>99% monomer conversion) | 21 | 95 |
|  | 2650 | 0.4 |  |  | 2540 | 0.4 |  |  | 159 | 5 |
| 2 min | 26 | 100 |  | 2 min | 25 | 99.3 |  | 2 min | 31 | 97.7 |
| 30 min | 27 | 99.4 |  |  | 2531 | 0.7 |  |  | 2521 | 2.3 |
|  | 2648 | 0.6 |  | 30 min | 48 | 100 |  | 30 min | 106 | 100 |
| 7 h | 104 | 100 |  | 7 h | 119 | 100 |  | 7 h | 91 | 100 |

**Table S5:** Size (volume) and %distribution of initial nanoparticle systems made at 20% solid concentration and their degraded nanoparticles under light.

(a) (b) (c)

| 50% MeOH | Size/nm | % |  | 60% MeOH | Size/nm | % |  | 75% MeOH | Size/nm | % |
| --- | --- | --- | --- | --- | --- | --- | --- | --- | --- | --- |
| **Initial**  (~21% monomer conversion) | 8 | 98 |  | **Initial**  (100% monomer conversion) | 86 | 100 |  | **Initial**  (100% monomer conversion) | 27 | 98.6 |
|  | 57 | 1 |  | **2 min** | 84 | 100 |  |  | 2338 | 1.4 |
|  | 133 | 1 |  | **30 min** | 84 | 100 |  | **2 min** | 28 | 99.3 |
| **2 min** | 19 | 32 |  | **7 h** | 84 | 100 |  |  | 2485 | 0.7 |
|  | 48 | 23 |  | **24 h** | 103 | 100 |  | **30 min** | 33 | 96.8 |
|  | 163 | 41 |  |  |  |  |  |  | 2349 | 3.2 |
| **30 min** | 56 | 100 |  |  |  |  |  | **7 h** | 184 | 92.4 |
| **7 h** | 88 | 92 |  |  |  |  |  |  | 2645 | 7.6 |
|  | 2237 | 8 |  |  |  |  |  | **24 h** | 180 | 100 |
| **24 h** | 155 | 100 |  |  |  |  |  |  |  |  |
| (d) |  |  |  |  |  |  |  |  |  |  |
| 50% MeOH | Size/nm | % |  |  |  |  |  |  |  |  |
| **Initial**  (100% monomer conversion) | 621 | 100 |  |  |  |  |  |  |  |  |
| **2 min** | 402 | 100 |  |  |  |  |  |  |  |  |
| **30 min** | 587 | 100 |  |  |  |  |  |  |  |  |
| **7 h** | 594 | 100 |  |  |  |  |  |  |  |  |
| **24 h** | 359 | 100 |  |  |  |  |  |  |  |  |

**Table S6:** Size (volume) and %distribution of initial nanoparticle systems made at 30% solid concentration and their degraded nanoparticles under light.

(a) (b) (c)

| 50% MeOH | Size/nm | % |  | 60% MeOH | Size/nm | % |  | 75% MeOH | Size/nm | % |
| --- | --- | --- | --- | --- | --- | --- | --- | --- | --- | --- |
| **Initial**  (~16% monomer conversion) | 1.79 | 100 |  | **Initial**  (100% monomer conversion) | 261 | 100 |  | **Initial**  (100% monomer conversion) | 182 | 100 |
| **2 min** | 9 | 96.5 |  | **2 min** | 279 | 100 |  | **2 min** | 182 | 100 |
|  | 28 | 3.5 |  | **30 min** | 229 | 100 |  | **30 min** | 173 | 100 |
| **30 min** | 34 | 65 |  | **7 h** | 213 | 100 |  | **7 h** | 152 | 100 |
|  | 129 | 35 |  | **24 h** | 204 | 100 |  | **24 h** | 154 | 100 |
| **7 h** | 52 | 53 |  |  |  |  |  |  |  |  |
|  | 145 | 47 |  |  |  |  |  |  |  |  |
| **24 h** | 167 | 82 |  |  |  |  |  |  |  |  |
|  | 37 | 18 |  |  |  |  |  |  |  |  |
| (d) |  |  |  |  |  |  |  |  |  |  |
| 50% MeOH | Size/nm | % |  |  |  |  |  |  |  |  |
| **Initial**  (100% monomer conversion) | 372 | 100 |  |  |  |  |  |  |  |  |
| **2 min** | 267 | 100 |  |  |  |  |  |  |  |  |
| **30 min** | 203 | 100 |  |  |  |  |  |  |  |  |
| **7 h** | 207 | 100 |  |  |  |  |  |  |  |  |
| **24 h** | 288 | 100 |  |  |  |  |  |  |  |  |

**Table S7:** Initial morphology, conversion, *M_n_*, and *M_w_/M_n_* of the samples made with different solid and methanol compositions.

| **System** | **Conv.** | **Initial** | | **Initial Morphology** |
| --- | --- | --- | --- | --- |
|  |  | ***M_n_*** | ***M_w_/M_n_*** |  |
| **10% Solid** |  |  |  |  |
| 75% MeOH | >95% | 29500 | 1.47 | Spheres |
| 60% MeOH | >95% | 30000 | 1.50 | Spheres |
| 50% MeOH | >95% | 26400 | 1.49 | Spheres |
|  |  |  |  |  |
| **20% Solid** |  |  |  |  |
| 75% MeOH | >95% | 30500 | 1.44 | Spheres |
| 60% MeOH | >95% | 28400 | 1.38 | Vesicles |
| 50% MeOH | ~20% | 11900 | 1.13 | Worms |
| 50% MeOH | >95% | 33300 | 1.47 | Vesicles |
|  |  |  |  |  |
| **30% Solid** |  |  |  |  |
| 75% MeOH | >95% | 27700 | 1.34 | Vesicles |
| 60% MeOH | >95% | 28600 | 1.37 | Vesicles |
| 50% MeOH | >95% | 28500 | 1.38 | Vesicles |
| 50% MeOH | ~16% | 9300 | 1.14 | Worms (exhibits highly branched network with swollen junctions) |

**Table S8:** *M_n_* and *M_w_/M_n_* of the sample with undegraded nanoparticles (at 10% solid concentration and 60% MeOH) and the samples with degraded nanoparticles under 310 nm (Intensity=2.3± 0.35 mW/cm^2^).

|  | **Time** | ***M_n_*** | ***M_w_/M_n_*** |
| --- | --- | --- | --- |
|  | 0 | 32400 | 1.32 |
|  | 2 min | 30700 | 1.38 |
|  | 30 min | 26300 | 1.44 |
|  | 7 h | 7600 | 2.19 |

**Figure S10:** Evolution of normalized molecular weight distributions at different degradation times (2 min, 30 min, and 7 h). A nanoparticle system was made with 60% MeOH as the solvent and at 10 w/w% solid concentration. Degradation under 310 nm (Intensity=2.3± 0.35 mW/cm^2^).

**Figure S11:** TEM images of sphere nanoparticles after RAFT-PISA polymerization (at ~100% monomer conversion) and photodegradation. Polymerization conditions: Molar ratio of PVK:poly(HPMA):VA-044=400:1:0.2 in 60% MeOH as the solvent, at 10 w/w% solid concentration, and 50 °C. Degradation conditions: under 310 nm (Intensity=2.3± 0.35 mW/cm^2^) at different time=2 min, 30 min, and 7 h.

**Table S9:** *M_n_* and *M_w_/M_n_* of the sample with undegraded nanoparticles (at 10% solid concentration and 50% MeOH) and the samples with degraded nanoparticles under 450 nm (Intensity=1.9±0.20 mW/cm^2^).

| **Time/h** | ***M_n_*** | ***M_w_/M_n_*** |  |
| --- | --- | --- | --- |
| 0 | 28900 | 1.40 |  |
| 1 | 28000 | 1.44 |  |
| 5 | 28600 | 1.39 |  |
| 7 | 29700 | 1.33 |  |
| 24 | 28700 | 1.39 |  |

**Figure S12:** Evolution of normalized molecular weight distributions at different degradation times (1 h, 5 h, 7 h, and 24 h). A nanoparticle system was made with 50% MeOH as the solvent and at 10 w/w% solid concentration. Degradation under 450 nm (Intensity=1.9±0.20 mW/cm^2^).

**
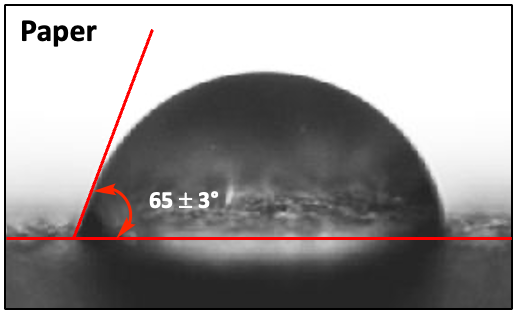
**

**Figure S13:** Contact angle measurements of water on unmodified paper.

**Table S10:** Contact angle measurements of water on polymer nanoparticle coatings on paper. (a) Water drops on undegraded and degraded sphere coatings on paper. (b) Water drops on undegraded and degraded worm coatings on paper. (c) Water drops on unmodified paper.

(a) (b) (c)

| **Spheres** | |  | **Worms** | |  | **Paper** |
| --- | --- | --- | --- | --- | --- | --- |
| Contact angle (°) | Contact angle (°) |  | Contact angle (°) | Contact angle (°) |  | Contact angle (°) |
| (Undegraded coatings) | (Degraded coatings) |  | (Undegraded coatings) | (Degraded coatings) |  | 60.3 |
| 51.0 | 54.5 |  | 54.4 | 59.1 |  | 66.8 |
| 51.5 | 54.3 |  | 55.5 | 57.3 |  | 61.2 |
| 49.0 | 53.5 |  | 54.9 | 58.5 |  | 61.8 |
| 52.2 | 53.9 |  | 54.5 | 56.5 |  | 64.0 |
| 50.6 | 55.1 |  | 53.2 | 57.7 |  | 64.3 |
| 49.5 | 56.4 |  | 53.6 | 57.1 |  | 65.2 |
|  |  |  |  |  |  | 67.4 |
|  |  |  |  |  |  | 69.4 |
|  |  |  |  |  |  | 63.8 |
|  |  |  |  |  |  | 67.9 |
|  |  |  |  |  |  | 68.0 |

**
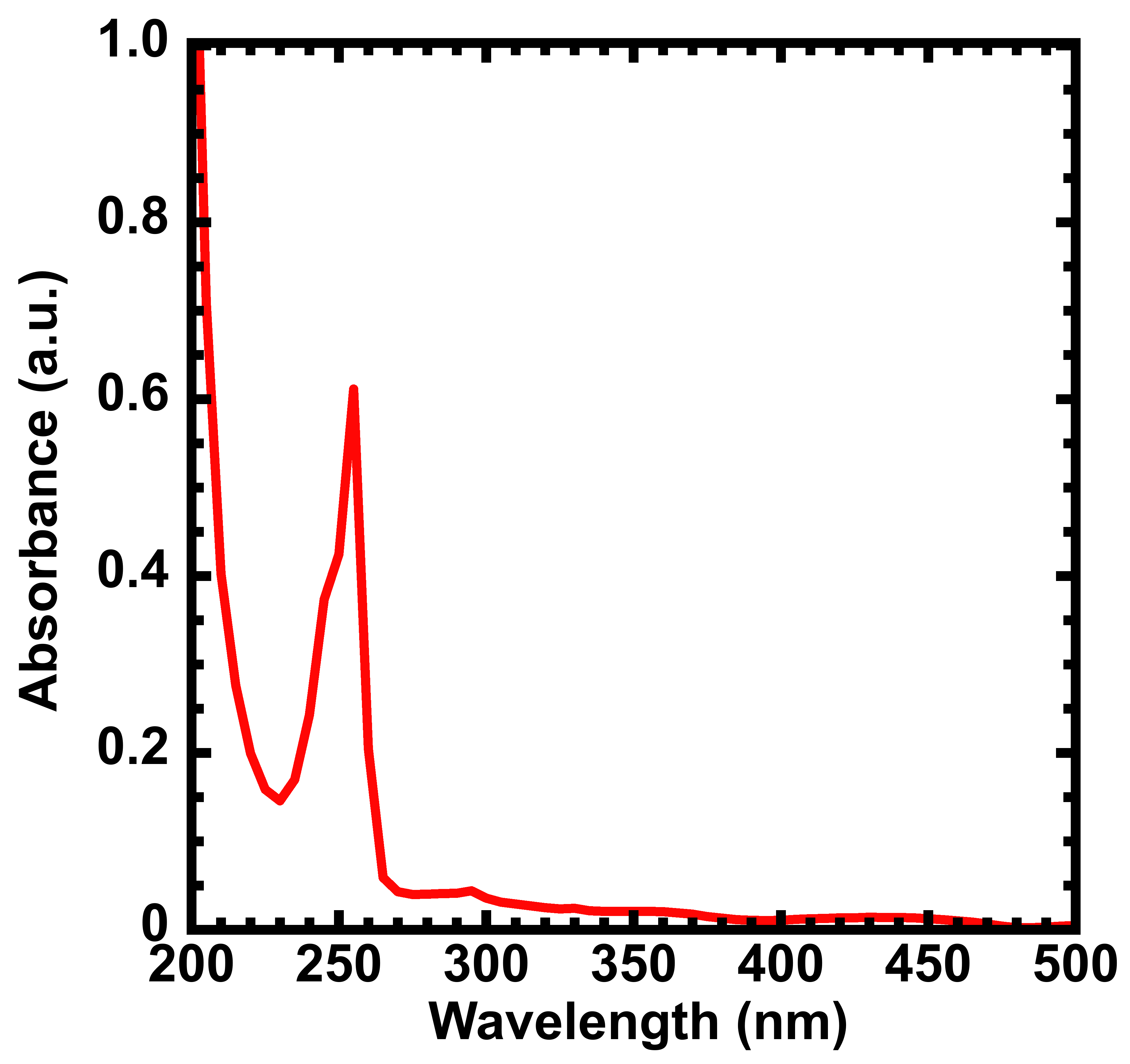
**

**Figure S14:** Absorbance spectrum of 9-fluorenone dye in methanol.


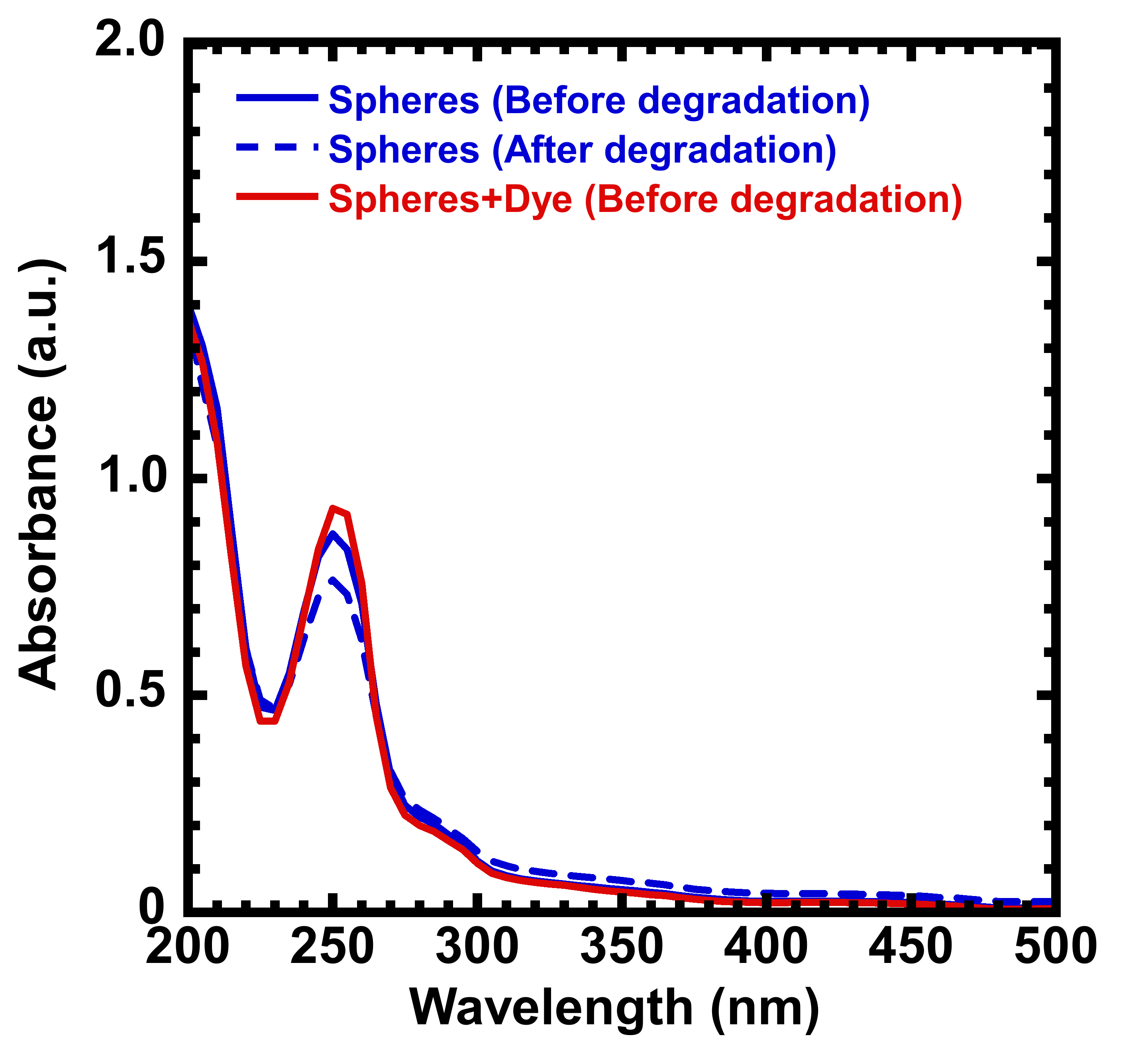


**Figure S15:** Absorbance spectra of degraded and undegraded sphere nanoparticles and sphere nanoparticles encapsulated with 9-fluorenone dye molecules (before degradation). Dye encapsulation: 500 μL of sphere nanoparticles made with 60% MeOH was mixed with 50 μL of 9-fluorenone dye in methanol. Degradation conditions: under 350 nm (Intensity=2.5± 0.13 mW/cm^²^) for 24 h.

**Figure S16:** TEM images of sphere nanoparticles before and after encapsulation. Synthesis of spheres- RAFT-PISA polymerization (~100% monomer conversion), Molar ratio of PVK:poly(HPMA):VA-044=400:1:0.2 in 60% MeOH as the solvent, at 10 w/w% solid concentration, and 50 °C. Dye encapsulation: 500 μL of sphere nanoparticles made with 60% MeOH was mixed with 50 μL of 9-fluorenone dye in methanol.

**References**

[1] K. G. E. Bradford, R. D. Gilbert, M. A. S. N. Weerasinghe, S. Harrisson, D. Konkolewicz, *Macromolecules* **2023**, *56*, 8784-8795.

[2] M. A. S. N. Weerasinghe, P. A. McBeth, M. C. Mancini, I. O. Raji, P. M. Needham, K. Yehl, Z. Oestreicher, D. Konkolewicz, *Chemical Engineering Journal* **2024**, *483*, 149307.

[3] X. Pan, F. Zhang, B. Choi, Y. Luo, X. Guo, A. Feng, S. H. Thang, *Eur. Polym. J.* **2019**, *115*, 166–172.
